# Liver Transplantation for Unresectable Intrahepatic Cholangiocarcinoma: The Role of Sequencing Genetic Profiling

**DOI:** 10.3390/cancers13236049

**Published:** 2021-12-01

**Authors:** Salvatore Gruttadauria, Floriana Barbera, Duilio Pagano, Rosa Liotta, Roberto Miraglia, Marco Barbara, Maria Grazia Bavetta, Calogero Cammà, Ioannis Petridis, Daniele Di Carlo, Pier Giulio Conaldi, Fabrizio Di Francesco

**Affiliations:** 1 Department for the Treatment and Study of Abdominal Diseases and Abdominal Transplantation, Istituto di Ricovero e Cura a Carattere Scientifico-Istituto Mediterraneo per i Trapianti e Terapie ad Alta Specializzazione (IRCCS-ISMETT), University of Pittsburgh Medical Center Italy (UPMC Italy), 90127 Palermo, Italy; dpagano@ismett.edu (D.P.); ipetridis@ismett.edu (I.P.); fdifrancesco@ismett.edu (F.D.F.); 2 Department of General Surgery and Medical-Surgical Specialties, University of Catania, 95123 Catania, Italy; 3 Laboratorio di Patologia Clinica, Microbiologia e Virologia, Dipartimento di Medicina di Laboratorio e Biotecnologie Avanzate, Istituto di Ricovero e Cura a Carattere Scientifico-Istituto Mediterraneo per i Trapianti e Terapie ad Alta Specializzazione (IRCCS-ISMETT), University of Pittsburgh Medical Center Italy (UPMC Italy), 90127 Palermo, Italy; fbarbera@ismett.edu (F.B.); ddicarlo@ismett.edu (D.D.C.); 4 Pathology Unit, Department of Diagnostic and Therapeutic Services, Istituto di Ricovero e Cura a Carattere Scientifico-Istituto Mediterraneo per i Trapianti e Terapie ad Alta Specializzazione (IRCCS-ISMETT), University of Pittsburgh Medical Center Italy (UPMC Italy), 90127 Palermo, Italy; rliotta@ismett.edu; 5 Radiology Service, Department of Diagnostic and Therapeutic Services, Istituto di Ricovero e Cura a Carattere Scientifico-Istituto Mediterraneo per i Trapianti e Terapie ad Alta Specializzazione (IRCCS-ISMETT), University of Pittsburgh Medical Center Italy (UPMC Italy), 90127 Palermo, Italy; rmiraglia@ismett.edu; 6 Research Department, Istituto di Ricovero e Cura a Carattere Scientifico-Istituto Mediterraneo per i Trapianti e Terapie ad Alta Specializzazione (IRCCS-ISMETT), University of Pittsburgh Medical Center Italy (UPMC Italy), 90127 Palermo, Italy; mbarbara@ismett.edu (M.B.); pgconaldi@ismett.edu (P.G.C.); 7 Unit of Hepatic Oncology, Division of Internal Medicine 2, Azienda Ospedaliera Ospedali Riuniti Villa Sofia-Cervello, 90146 Palermo, Italy; mariagraziabavetta@gmail.com; 8 Section of Gastroenterology & Hepatology, Department of Health Promotion, Mother and Child Care, Internal Medicine and Medical Specialties (PROMISE), University of Palermo, 90127 Palermo, Italy; calogero.camma@unipa.it

**Keywords:** intrahepatic cholangiocarcinoma, liver transplantation, next-generation sequencing

## Abstract

**Simple Summary:**

Intrahepatic cholangiocarcinoma is a rare disease with increasing incidence and mortality still characterized by an insufficient clinical outcome. Growing attention has recently surrounded this disease, and liver transplantation has emerged as a novel curative treatment for cholangiocarcinoma, along with a better understanding of genetic alterations potentially capable of driving tumorigenesis. The aim of this paper is to present a clinical description of our case series of patients affected by intrahepatic cholangiocarcinoma and by mixed forms of hepatocellular and cholangiocellular carcinoma, together with a genomic profiling of mutations occurring in a panel of genes relevant to solid tumor cancer investigations. Mutations were observed in genes activating signaling pathways known to be involved in intrahepatic cholangiocarcinoma tumorigenesis; a strong association was observed between mutation in genes involving the Notch signaling pathway and tumor size (point-biserial *rho_pb_* = 0.93).

**Abstract:**

Intrahepatic cholangiocarcinoma (iCCA) is a rare and aggressive primary liver tumor, characterized by a range of different clinical manifestations and by increasing incidence and mortality rates even after curative treatment with radical resection. In recent years, growing attention has been devoted to this disease and some evidence supports liver transplantation (LT) as an appropriate treatment for intrahepatic cholangiocarcinoma; evolving work has also provided a framework for better understanding the genetic basis of this cancer. The aim of this study was to provide a clinical description of our series of patients complemented with Next-Generation Sequencing genomic profiling. From 1999 to 2021, 12 patients who underwent LT with either iCCA or a combined hepatocellular and cholangiocellular carcinoma (HCC-iCCA) were included in this study. Mutations were observed in gene activating signaling pathways known to be involved with iCCA tumorigenesis (KRAS/MAPK, P53, PI3K-Akt/mTOR, cAMP, WNT, epigenetic regulation and chromatin remodeling). Among several others, a strong association was observed between the Notch pathway and tumor size (point-biserial *rho_pb_* = 0.93). Our results are suggestive of the benefit potentially derived from molecular analysis to improve our diagnostic capabilities and to devise new treatment protocols, and eventually ameliorate long-term survival of patients affected by iCCA or HCC-iCCA.

## 1. Introduction

Intrahepatic cholangiocarcinoma (iCCA) is a relatively rare and highly aggressive form of primary liver tumor, characterized by increasing incidence and mortality rates worldwide, which have given rise to growing scientific interest aimed at better disease classification, molecular diagnostics, and pathology accuracy [1,2,3,4,5,6,7]. Though radical surgery was once considered the only curative option, considerable efforts have been made in search of protocols and therapeutic schemes to improve the outcome and survival rate in selected patients undergoing liver transplantation (LT), now being considered an appropriate treatment option for cholangiocarcinoma patients [8,9,10,11,12].

Although the majority of iCCA patients have no underlying liver disease and the clinical presentation is non-specific, most of the risk factors for hepatocellular carcinoma (HCC) have been discovered to be risk factors for iCCA as well, including cirrhosis, chronic viral hepatitis, excessive alcohol consumption, diabetes, and obesity, thus supporting the hypothesis of common pathobiological pathways to all primary liver parenchymal tumors. This would explain why mixed HCC and iCCA forms (HCC-iCCA) are increasingly evident in the anatomopathological examinations of native livers among transplant series [5,13].

Few data are available about the incidence and post-transplant outcomes of HCC-iCCA and iCCA in patients undergoing transplantation, usually misdiagnosed and treated as HCC [13,14]. Diagnosis is rarely made preoperatively because radiological features are hardly distinguishable from other primary liver tumors, due to the atypical enhancing patterns, the small size of each component, and because the tumors are composed of intermediate cells [15,16].

Mixed HCC-iCCA lesions have worse outcomes following LT than patients with HCC with a reported five-year recurrence rate of 65% [17,18,19]. These data impose the need to devise pre-transplant differential diagnostic systems. A pre-operative discrimination could be useful for redirecting these patients towards research protocols combining adjuvant or neoadjuvant therapy for iCCA in the transplant setting. 

From a pathogenic point of view, the large heterogeneity and the rare occurrence of iCCA, together with reduced knowledge of its molecular mechanisms, are features contributing to the difficulties in finding an appropriate cure [6,20]. Moreover, iCCA is usually diagnosed at an advanced stage, is non-responsive to chemotherapy and surgical treatment, and minor progresses have been realized concerning the development of treatments for this tumor [21]. In recent years, however, a massive on-going evolving work has provided a framework for better understanding the genetic basis of this cancer and identifying genetic alterations that drive tumorigenesis [22,23]. Signaling pathways, drivers of carcinogenesis and potential targets for therapies in iCCA, include KRAS/MAPK, EGFR, IL-6/STAT, IDH1/2, FGFR2 and MET signaling. No oncogenic addiction loops have been described so far, and molecular classification of iCCA based on gene signatures or molecular abnormalities is not ready for clinical application. Immunostaining to discover markers of iCCA or progenitor cell features should be detected to distinguish iCCA or mixed hepatocellular-cholangiocarcinoma tumors with the aim of changing clinical management.

Diagnosis of iCCA is difficult; thus, a noninvasive approach towards assessing and monitoring the tumor-specific mutational profile is desirable to improve diagnosis and to tailor treatment.

The aim of the present study is to describe our case series of patients affected by iCCA or HCC-iCCA, complementing clinical information with Next-Generation Sequencing (NGS) genomic profiling, to examine somatic mutations in genes involved in tumor progression and attempt to associate pathogenic alterations in specific signaling pathways to clinical and pathological presentation of the disease.

## 2. Materials and Methods

### 2.1. Study Population 

All adult patients who underwent liver transplantation at our institution from 1999 to 2021 were retrospectively screened to identify those with histopathological findings of iCCA or HCC-iCCA, either at pre-transplant biopsy or incidentally at pathological examination of the explanted liver. Twelve cases were then selected for this study. While 10 of the 12 cases were incidentally diagnosed on pathologic examination, two recent transplants were performed for biopsy-proven locally advanced unresectable iCCA [1]. Notably, six (50%) of these transplantations were performed in the last three years. The study was conducted according to the guidelines of the Declaration of Helsinki and approved by the ISMETT Ethics Committee (Study ID: IRRB/42/16, date of approval: 23 June 2017).

### 2.2. Genomic DNA Extraction 

QIAamp DNA FFPE Tissue kit (Qiagen, Germantown, MD, USA) was used to extract DNA from 10 unstained slides. The quality of samples was evaluated by Genomic DNA ScreenTape System (4200 TapeStation System, Agilent Technologies, Santa Clara, CA, USA), the purity by NanoDrop Spectrophotometer (Thermo Fisher Scientific, Waltham, MA, USA), and concentration by Qubit dsDNA BR Assay kit (Thermo Fisher Scientific, Waltham, MA, USA).

### 2.3. Next-Generation Sequencing Analysis

Library construction was performed using 200 ng of genomic DNA input and SureSelect XT HS protocol (Agilent Technologies), based on the enzymatic fragmentation of DNA (SureSelect Enzymatic fragmentation kit, Agilent Technologies) and subsequent probe-mediated hybridization capture (target enrichment). The study was performed using a targeted NGS solution, SureSelect Cancer All-In-One (AIO) Solid Tumor Assay (Agilent Technologies), which allows for the detection of variants in 98 genes relevant for solid tumor cancer investigations (Supplementary Table S1). Sequencing 2 × 150 PE (paired-end) of an equimolar library was performed by the MiSeq (Illumina, San Diego, CA, USA). Quality check of raw reads was realized with the FastQC tool [24]. Alissa Align and Call software (Agilent Technologies) were used for sequence alignment (human genome reference hg38), index and primer trimming, and variant call. Analysis of tumor-related genes was achieved with Alissa Interpret software (Agilent Technologies) using the following criteria: PASS filter, frequency of alternative (Alt) allele (versus reference allele, Ref) ≥ 3%, variant call quality = 100 and read depth > 100. The SSEL Cancer AIO Solid Tumor Assay can detect somatic alterations with VAF ≥ 5%. All variants were evaluated to remove synonymous variants, leaving only variants affecting coding sequences (missense, InDel/frameshift, stop gained, stop lost, initiator codons, in-frame insertions, in-frame deletions, splice/intronic variants). The variants were evaluated according to ClinVar Classification, COSMIC database, in silico prediction tools (Protean, PolyPhen2, LRT and Mutation Assessor). Finally, each candidate variant’s interpretation was confirmed using the VarSome tool [25].

### 2.4. Statistical Analysis

Categorical variables are presented as frequency and percentage. Numerical variables are presented as median, interquartile range (IQR) and, optionally, range. Associations between clinical or pathological characteristics and the presence of a mutation in a specific gene (or in one of the genes involved in a biochemical pathway) are measured by means of either the *phi* mean square contingency coefficient or by means of the *rho_pb_* point-biserial correlation coefficient, as appropriate.

## 3. Results

### 3.1. Preoperative Clinical Data 

From 30 July 1999 to 30 September 2021, a total of 1207 LTs have been performed at our institution on adult patients, 548 of which were on patients with a tumor diagnosis, but only 12 patients were transplanted with either iCCA or a mixed form HCC-iCCA. More specifically, patients selected for this study are of three different subtypes: (1) 5 patients whose diagnosis was incidentally established on pathologic examination as HCC-iCCA; (2) 5 patients whose diagnosis was incidentally established on pathologic examination as iCCA; (3) 2 patients (one iCCA and one HCC-iCCA) whose biopsy-proven diagnosis was established before transplant. Patients in the first two groups fulfilled the criteria for transplantability (being either misdiagnosed as HCC or as end-stage liver disease) and were consequently put in waiting list for LT, which was considered the best therapeutic option. Of the 2 patients who were properly diagnosed before LT as HCC-ICCA and iCCA, respectively, one was affected by a 8 cm diameter cholangiocarcinoma lesion infiltrating the three suprahepatic veins and imprinting the vena cava, the other was affected by a 8 cm centrally located cholangiocarcinoma lesion with infiltration of the hilar plate and the left portal branch. Both were judged as unresectable and redirected to selective internal radiation therapy (SIRT) and neoadjuvant chemotherapy before LT (Table 1). Incidentally diagnosed HCC-iCCA and iCCA groups of patients were characterized by similar clinical characteristics, both showing a high prevalence of male sex, similar age distributions (median (IQR) of 60 (55–60) and 62 (58–65), respectively) and relatively similar distributions of liver disease etiologies (Table 1). All patients in both groups had liver cirrhosis. Patients in the iCCA group presented with slightly better preserved liver function (with a MELD of 10 (9–11) vs. 14 (11–15) of the HCC-iCCA group). Only two patients in the iCCA group underwent mini-invasive HCC-aimed curative treatments (l hepatic resection and 1 microwave thermal ablation, respectively). Two patients (40%) in the iCCA group and three patients (60%) in the HCC-iCCA group underwent transarterial chemo-embolization before LT (Table 1).

Anatomopathological findings showed that patients in the iCCA group were characterized by a slightly higher tumor size than patients affected by HCC-iCCA (1.8 (1.6–2.5) vs. 2.5 (2.0–3.0), respectively). Interestingly, in all cases in which we observed a coexistence of cholangiocarcinoma (either HCC-iCCA or iCCA) with HCC-only nodules, CCA nodules were always the largest ones. These data could assume relevance in clinical practice if confirmed in larger cohorts.

Of the two non-incidentally diagnosed patients, one was a 67-year-old male affected by a 8 cm HCC-iCCA developed on a non-cirrhotic liver, with extensive vascular invasion; the other was a 65-year-old female affected by a 8 cm iCCA lesion developed on a cirrhotic liver; MELD score were 5 and 9, respectively. They were both treated with transarterial radio-embolization and noeadjuvant chemotherapy before transplant with a partial response.

### 3.2. Patient Outcome

Two of the 12 recruited patients died, respectively belonging to the iCCA and HCC-iCCA incidentally diagnosed patient. The iCCA patient died at 76th post-operative day because of a recurrence of HCV infection; the HCC-iCCA patient underwent retransplantation almost 6 years after the first LT due to HCV recurrence and died because of multi-organ failure approximately 1 month afterwards.

### 3.3. NGS Analysis

We performed next-generation sequencing analysis (NGS) to examine somatic mutations in 98 genes involved in tumor progression in 12 tumor tissues of resected iCCAs/HCC-iCCAs. Subsequent to bioinformatic analysis, we identified 372 variants, of which 70 were of uncertain significance and 92 pathogenic or likely pathogenic, according to the literature data (i.e., ClinVar and COSMIC) and bioinformatic prediction tools.

**Figure 1:**
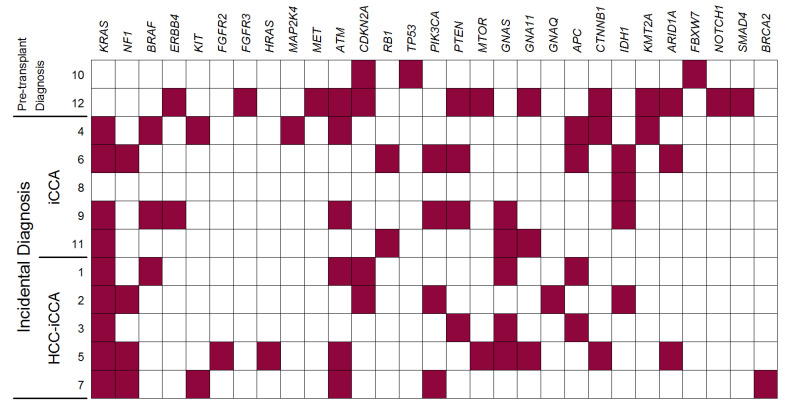
Genes presenting mutations in each of 12 patients affected by iCCA/HCC-iCCA who underwent liver transplantation (red box indicates the presence of mutation; white box indicates the absence of mutation).

Table 2 presents the distribution of the observed mutations by gene and the number of patients presenting at least one mutation for each gene. Genes are further categorized into the principal molecular pathways involved in iCCA (MAPK, P53, PI3K-Akt/mTOR, cAMP, WNT, epigenetic regulation, chromatin remodeling, NOTCH, TGFβ, DNA Repair). Figure 1 presents a graphical depiction of the distribution of observed mutations among patients. More detailed information can be found in Table S2.

In our cohort, the pathogenic variants were mainly frequent in the MAPK pathway, involved in signal transduction by converting extracellular stimuli into cellular responses including differentiation, survival, tumorigenesis, and inflammatory and stress responses. In particular, we found KRAS pathogenic variants in 9 patients at known hotspots of amino acid residues of the position 12, 13 and 61 (G12D, G12V and Q61H), which are related with a decrease in overall survival and progression-free survival in iCCA patients. Recent data indicate that liver cancer progression can be decreased by blocking or inhibiting dysregulated components of the MAPK signaling pathway [26]. Thus, some of these mutated proteins could become therapeutic targets for the treatment of iCCA [27]. 

Genetic alteration in genes involved in the P53 pathway were identified in 10 patients. Previous data indicated that the prevalence of TP53 alterations in iCCA patients varied considerably ranging from 10 to 40%, and were associated with a worse outcome in iCCA. Moreover, dysregulation of the P53 pathway in iCCA is responsible for changes in the composition and metabolism of cytotoxic biliary constituents that cause alterations in intracellular signaling cascades [28].

Pathogenic alteration in genes involved in the PI3K/Akt pathway were observed in 7 patients. This signaling pathway regulates cellular glucose metabolism and its dysregulation may have deleterious effects on normal cell metabolism. Mutations in PI3K/Akt are commonly identified in iCCA cells and control sensitivity of cells to G1 arrest induced by mTOR inhibitors. Recent data indicate that activation of the PI3K signaling pathway in iCCA cells may protect the cells from oxaliplatin-induced cytotoxicity [29]. Hence, chemotherapeutic agents might function better in treating cancer cells if the PI3K signaling pathway is blocked.

We found in 7 cases pathogenic alterations in genes related to the cAMP signaling pathway, which has been demonstrated to be important in the regulation of cholangiocyte proliferative events during damage and in physiologic conditions. In fact, the cholangiocytes are the main liver collectors of neuropeptides, angiogenic factor signals and hormones, and use cAMP as a second messenger [30].

Mutations in genes coding for proteins of the WNT signaling pathway were observed in 6 patients. Recent findings indicate the use of WNT inhibitors for increasing apoptosis, reducing tumor proliferation, and causing iCCA regression, and so are a potential therapeutic strategy for iCCA [31].

We identified in 4 patients genetic alterations in IDH1, involved in metabolic pathways, implicated in 10–23% of iCCA but not identified in HCC cases. IDH converts isocitrate to α-ketoglutarate and produces NADPH, involved in many cellular processes. Mutated IDH causes the loss of its enzymatic activity with a gain of a neomorphic function to produce the R-enantiomer of 2-hydroxyglutarate (R-2-HG), which is considered to be an oncometabolite [32].

Of note, we found in 3 patients pathogenic variants in ARID1A, which encodes a subunit of SWI/SNF protein complexes that regulate gene activity, by a process known as chromatin remodeling, which may be altered. Recent data report that mutation involving chromatin-modulating genes occurs in approximately 25–50% of iCCA cases [33].

### 3.4. Associations between Mutations and Clinical Characteristics of the Patients

**Figure 2:**
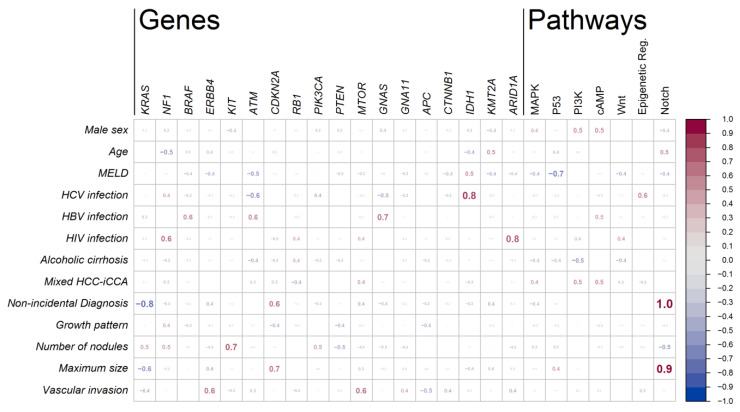
Association coefficients between mutations and clinical characteristics of the patients. Each cell represents the value (ranging from −1 to +1) of an association index between a clinical characteristic of the patient (in row) and the presence of at least one mutation observed in the gene (in column). The last seven columns refer to the presence of at least one mutation observed in the group of genes involved in a biochemical pathway. Font size is proportional to the absolute value of the coefficient. Genes and pathways with mutations observed in one patient only were excluded. Reported association indexes are *phi* mean square contingency coefficient or the *rho_pb_* point-biserial correlation coefficient, as appropriate.

As briefly summarized in Figure 2, the strongest positive associations were found between mutations in the Notch pathway and both (1) the size of the cholangiocarcinoma nodule (*rho_pb_ =* 0.93) and (2) the non-incidental diagnosis (*rho_pb_ =* 1.00). This is due to the fact that both genes involved in the Notch pathway (*FBXW7* and *NOTCH1*) and the present mutations only concern the two pre-transplant diagnosed patients (see also Figure 1). Since these two patients also present by far the two biggest nodules, the association of Notch with tumor size is possibly the one with more clinical significance. Also interesting in this respect is the strong negative association between the non-incidental diagnosis and the *KRAS* gene (*phi* = −0.77), which indeed present mutations only in the incidentally diagnosed groups (Figure 1). Maximum nodule size also appears to have a moderate association with *CDKN2A* (*rho_pb_ =* 0.66). Other relatively strong positive associations are found between liver disease etiology and genes *IDH1* and *ARID1A* (*phi* = 0.82 and *phi* = 0.77 for HCV and HIV infection, respectively), and a moderate association was also observed between HBV infection and *GNAS* (*phi* = 0.68). Interestingly, a moderate negative association was observed between the P53 pathway and MELD (*rho_pb_* = −0.67), resulting from the relatively higher MELD of the two patients not showing any mutation in the P53 pathway.

## 4. Discussion

Surgery remains the best treatment available for iCCA. However, the survival rate is poor. Ideally, liver resection and regional lymphadenectomy are the best treatments, though they are connoted by a recurrence-free survival not higher than 39% 5 years after surgery [34,35,36]. Alternatively, systemic chemotherapy and loco-regional therapy such as SIRT do offer a minimal response, with a progression-free survival ranging from 11 to 20 months [37,38].

In this regard, liver transplantation, which had previously been contraindicated for iCCA and HCC-iCCA patients in view of the poor outcome reported, has recently emerged as a viable treatment strategy in selected patients after the relatively favorable outcome reported by two Spanish retrospective studies [17,18], which found a five-year survival rate of 65% in cirrhotic patients with small (<2 cm) iCCAs (or HCC-iCCAs) incidentally discovered on pathological examination of the native liver. Another impressive finding of 83% 5-year survival was reported by a small series of six patients with locally advanced iCCAs transplanted after achieving disease stability after neoadjuvant chemotherapy [19]. While these results are promising, they are difficult to interpret due to pathological heterogeneity and they are limited by the fact that patients are often misdiagnosed and treated as HCC. Therefore, it would be favorable to better discriminate patients both between HCC and iCCA and between iCCAs and mixed-form HCC-iCCAs in order to redirect these patients towards proper therapeutic protocols. 

In this regard, a better understanding of the molecular pathogenic complexity could be useful to devise new diagnostic and targeted therapeutical strategies. A recent study on genotyping of circulating tumor DNA in CCA patients revealed diagnostic and prognostic information helpful in facilitating diagnosis and personalizing and adapting therapeutic strategies [17]. Given the emerging data regarding actionable targets for treating iCCA, molecular testing of metastatic and unresectable tumors should be considered, aimed at allowing preventive and therapeutic measures available based upon the cancer genes identified in an individual [9,28]. 

With this study, we presented our series of iCCA and HCC-iCCA patients who underwent LT, and who were retrospectively analyzed in order to explore potential discriminative patterns between both clinical characteristics and genomic variants in search of potential associations between them.

From a clinical point of view, as shown in Table 1, our series of patients, despite being small, is characterized by a marked amount of variability in both etiology and liver function. As has been previously and repeatedly reported, we also noticed how challenging the diagnostic process can be for these patients with only 9 patients out of 12 with radiological evidence of liver tumor and only 2 with a correct diagnosis of iCCA or HCC-iCCA. On the other hand, our molecular analysis confirmed the presence of pathogenic alterations in specific signaling pathways involved in aberrant modulations of cell cycle, apoptosis and proliferation. Our results also confirmed the great genetic heterogeneity of iCCA, made evident by the presence of many genomic alterations which might be associated with the specific context in which the tumor grows. In particular, genetic variants in CDKN2A, KRAS and TP53 were found to be independent prognostic factors in iCCA, even after taking into account several pathologic and clinical variables, treatments and disease stages [39].

Some interesting associations emerged between mutation occurrence and clinical manifestation of the disease, namely a strong association (*rho_pb_* = 0.93) between cholangiocarcinoma nodule size and the Notch pathway, and between liver disease etiology and genes IDH1 and ARID1A (*phi* = 0.82 and *phi* = 0.77 for HCV and HIV infection, respectively).

We do acknowledge that this study has several limitations. The small sample size, in particular, limits the generalizability of our findings to the potentially transplantable iCCA/HCC-iCCA population; moreover, its retrospective nature focused only on actually transplanted patients instead of potentially transplantable ones, making it impossible to select appropriate control groups with different indications. 

However, even in the presence of these limitations, we perceive that our findings provide suggestive evidence that research on clinical and molecular biomarkers should be boosted when in search of new therapeutic protocols combining oncologic medical treatments with hepatic resection and liver transplantation, to ameliorate the standard of care and eventually the long-term survival of patients affected by iCCA/HCC-iCCA. Future controlled prospective studies on larger cohorts could also be conducted with a better understanding of iCCA heterogeneity and stratification of iCCA subtypes [40].

## 5. Conclusions

Molecular analysis confirmed many of the findings already reported in the literature with observed mutations in genes activating signaling pathways well known to be involved with iCCA tumorigenesis. An interestingly strong association was found between tumor size and the occurrence of mutations in the Notch pathway (*rho_pb_* = 0.93). 

In line with previously published data, our analysis on the tumor tissue of iCCA-transplanted patients suggests that further studies in this direction could lead to encouraging results and supports the potential prognostic value of NGS-based personalized therapies and the translation of molecular analysis into oncology practice.

## Figures and Tables

**Table 1:** Clinical and pathological characteristics of 12 patients transplanted with HCC-iCCA or iCCA.

	Incidentally Diagnosed on Pathologic Examination		Pre-Transplant Diagnosed
	HCC-iCCA	iCCA	
N	5	5	2
Male sex	5 (100)	4 (80)	1 (50)
Age, median (IQR) ^§^	60 (55–60)	62 (58–65)	65, 67 ^§^
MELD, median (IQR) ^§^	14 (11–15)	10 (9–11)	9, 6 ^§^
Waiting list time, days ^§^	132 (93–147)	177 (51–186)	4, 114 ^§^
**Etiology of liver disease**			
HCV infection	1 (20)	1 (20)	0 (0)
HBV infection	1 (20)	1 (20)	0 (0)
HCV-HIV co-infection	0 (0)	1 (20)	0 (0)
HBV-HIV co-infection	1 (20)	0 (0)	0 (0)
Alcoholic cirrhosis	0 (0)	1 (20)	0 (0)
Unknown etiology	2 (40)	1 (20)	2 (100)
**Pretransplant oncologic diagnosis**			
Hepatocellular carcinoma	4 (80)	4 (80)	0 (0)
Intrahepatic cholangiocarcinoma	0 (0)	0 (0)	2 (100)
No liver tumor	1 (20)	1 (20)	
Liver cirrhosis	5 (100)	5 (100)	1 (50)
Radiological diagnosis of liver tumor	4 (80)	4 (80)	2 (100)
Radiological evidence of lymph nodes enlargement	5 (100)	3 (60)	2 (100)
**Previous treatments**			
Laparoscopic hepatic resection	0 (0)	1 (20)	0 (0)
Laparoscopic microwave thermal ablation	0 (0)	1 (20)	0 (0)
Transarterial chemo-embolization	3 (60)	2 (40)	0 (0)
Transartherial radio-embolization	0 (0)	0 (0)	2 (100)
Neoadjuvant chemotherapy	0 (0)	0 (0)	2 (100)
**Anatomopathological findings**			
Mixed HCC-iCCA	5 (100)	0 (0)	1 (50)
Macroscopic growth pattern			
Mass forming type	3 (60)	4 (80)	2 (100)
Intraductal growing type	2 (40)	0 (0)	0 (0)
Periductal infiltrating type	0 (0)	1 (20)	0 (0)
**Number of nodules**			
Monofocal tumor	1 (20)	0 (0)	2 (100)
2 nodules	2 (40)	4 (67)	0 (0)
3 nodules	0 (0)	0 (0)	0 (0)
4 nodules	2 (40)	1 (17)	0 (0)
Size of the greatest nodule, cm ^§^	1.8 (1.6–2.5)	2.5 (2.0–3.0)	Both 8 cm ^§^
Vascular invasion	1 (20)	2 (40)	1 (50)
Portal vein invasion	1 (20)	1 (20)	1 (50)
Hepatic vein invasion	1 (20)	1 (20)	1 (50)
Serous membrane	1 (20)	1 (20)	1 (50)

^§^ For the 2 pre-transplant diagnosed patients, quantitative variables are expressed as individual values instead of median (IQR).

**Table 2:** Distribution of observed pathogenic or likely pathogenic variants by gene, and number of patients presenting at least one mutation for each gene.

Pathway *GENE*	Mutations (N = 92)	Patients (N = 12)
**MAPK pathway**		
Overall	27 (29)	10 (83)
*KRAS*	10 (11)	9 (75)
*NF1*	5 (5)	4 (33)
*BRAF*	3 (3)	3 (25)
*ERBB4*	2 (2)	2 (17)
*KIT*	2 (2)	2 (17)
*FGFR2*	1 (1)	1 (8)
*FGFR3*	1 (1)	1 (8)
*HRAS*	1 (1)	1 (8)
*MAP2K4*	1 (1)	1 (8)
*MET*	1 (1)	1 (8)
**P53 pathway**		
Overall	17 (18)	10 (83)
*ATM*	10 (11)	6 (50)
*CDKN2A*	4 (4)	4 (33)
*RB1*	2 (2)	2 (17)
*TP53*	1 (1)	1 (8)
**PI3K-Akt/mTOR pathway**		
Overall	14 (15)	7 (58)
*PIK3CA*	8 (9)	4 (33)
*PTEN*	4 (4)	4 (33)
*MTOR*	2 (2)	2 (17)
**cAMP pathway**		
Overall	9 (10)	7 (58)
*GNAS*	5 (5)	5 (42)
*GNA11*	3 (3)	3 (25)
*GNAQ*	1 (1)	1 (8)
**Wnt pathway**		
Overall	9 (10)	6 (50)
*APC*	6 (7)	4 (33)
*CTNNB1*	3 (3)	3 (25)
**Epigenetic regulation**		
Overall	6 (7)	6 (50)
*IDH1*	4 (4)	*4 (33)*
*KMT2A*	2 (2)	*2 (17)*
**Chromatin remodeling**		
*ARID1A*	6 (7)	*3 (25)*
**Notch pathway**		
Any mutation	2 (2)	2 (17)
*FBXW7*	1 (1)	*1 (8)*
*NOTCH1*	1 (1)	*1 (8)*
**TGFβ pathway**		
*SMAD4*	1 (1)	1 (8)
**DNA Repair**		
*BRCA2*	1 (1)	1 (8)

## Data Availability

The data presented in this study are available on request from the corresponding author.

## Supplementary Materials

The following are available online at https://www.mdpi.com/article/10.3390/cancers13236049/s1, Table S1: Genes covered by SureSelect Cancer All-In-One (AIO) Solid Tumor Assay; Table S2: List of all pathogenic or likely-pathogenic mutations found in iCCA tissue samples from 12 patients who underwent liver transplantation.
