# Supplementary material for: Liver Transplantation for Unresectable Intrahepatic Cholangiocarcinoma: The Role of Sequencing Genetic Profiling
Source: Cancers (Basel). 2021 Dec 1;13(23):6049. doi: 10.3390/cancers13236049 (PMC8657183; doi:10.3390/cancers13236049)
Supplement: Supplementary file 1 [file cancers-13-06049-s001.zip › cancers-1458237-supplementary.pdf]

cancers MDPI

Supplementary Materials: Liver Transplantation for Unresectable Intrahepatic Cholangiocarcinoma: The Role of Sequencing Genetic Profiling

Salvatore Gruttadauria, Floriana Barbera, Duilio Pagano, Rosa Liotta, Roberto Miraglia, Marco Barbara, Maria Grazia Bavetta, Calogero Cammà, Ioannis Petridis, Daniele Di Carlo, Pier Giulio Conaldi and Fabrizio Di Francesco

Table S1. Genes covered by SureSelect Cancer All-In-One (AIO) Solid Tumor Assay.

ABL1	*CDKN2A*	*FGFR4*	*MLH1*	*RAF1*
AKT1	*CDKN2B*	*FOXL2*	*MSH2*	*RB1*
ALK	*CIC*	*GNA11*	*MSH6*	*RET*
APC	*CSF1R*	*GNAQ*	*MTOR*	*RIT1*
AR	*CTNNB1*	*GNAS*	*MYC*	*ROS1*
ARAF	*DDR2*	*HNF1A*	*MYCN*	*SMAD4*
ARID1A	*DNMT3A*	*HRAS*	*MYD88*	*SMARCB1*
ATM	*EGFR*	*IDH1*	*NF1*	*SMO*
BCL2	*ERBB2*	*IDH2*	*NF2*	*SRC*
BCR	*ERBB3*	*JAK2*	*NFE2L2*	*STK11*
BRAF	*ERBB4*	*JAK3*	*NOTCH1*	*TERT*
BRCA1	*ESR1*	*KDR*	*NRAS*	*TMPRSS2*
BRCA2	*ETV1*	*KIT*	*NTRK1*	*TP53*
CCND1	*ETV4*	*KMT2A*	*PDGFRA*	*TSC1*
CCND2	*ETV6*	*KRAS*	*PDGFRB*	*TSC2*
CCNE1	*EZH2*	*MAP2K1*	*PIK3CA*	*VEGFA*
CD274	*FBXW7*	*MAP2K2*	*PIK3R1*	*VHL*
CDH1	*FGFR1*	*MAP2K4*	*PTCH1*	*WT1*
CDK4	*FGFR2*	*MDM2*	*PTEN*	
CDK6	*FGFR3*	*MET*	*PTPN11*	

Table S2. List of all pathogenic or likely-pathogenic mutations found in iCCA tissue samples from 12 patients who underwent liver transplantation.

Gene (Transcript)	cDNA (cNomen)	Patient	Pathway
KRAS(NM_033360.4)	c.183A>C	1	MAPK
KRAS(NM_033360.4)	c.35G>A	2	MAPK
KRAS(NM_033360.4)	c.35G>T	3	MAPK
KRAS(NM_033360.4)	c.35G>T	4	MAPK
KRAS(NM_033360.4)	c.35G>A	5	MAPK
KRAS(NM_033360.4)	c.35G>T	6	MAPK
KRAS(NM_033360.4)	c.35G>A	7	MAPK
KRAS(NM_033360.4)	c.35G>A	9	MAPK
KRAS(NM_033360.4)	c.216G>A	9	MAPK
KRAS(NM_033360.4)	c.216G>A	11	MAPK

NF1(NM_001042492.2)	c.3313A>T	2	MAPK
NF1(NM_001042492.2)	c.3144G>T	2	MAPK
NF1(NM_001042492.2)	c.808C>T	5	MAPK
NF1(NM_001042492.2)	c.4273G>T	6	MAPK
NF1(NM_001042492.2)	c.5788T>C	7	MAPK
BRAF(NM_001374258.1)	c.144G>A	1	MAPK
BRAF(NM_001374258.1)	c.1039C>T	4	MAPK
BRAF(NM_001374258.1)	c.1531G>T	9	MAPK
ERBB4(NM_005235.3)	c.1818G>T	9	MAPK
ERBB4(NM_005235.3)	c.3902A>G	12	MAPK
KIT(NM_000222.2)	c.811C>T	4	MAPK
KIT(NM_000222.2)	c.2008A>T	7	MAPK
FGFR2(NM_022970.3)	c.2063G>A	5	MAPK
FGFR3(NM_001354809.2)	c.1849A>G	12	MAPK
HRAS(NM_176795.4)	c.34G>A	5	MAPK
MAP2K4(NM_001281435.2)	c.779G>A	4	MAPK
MET(NM_000245.4)	c.3629G>A	12	MAPK
ATM(NM_000051.3)	c.283C>T	1	P53
ATM(NM_000051.3)	c.6448G>A	1	P53
ATM(NM_000051.3)	c.7472G>T	4	P53
ATM(NM_000051.3)	c.8254A>T	4	P53
ATM(NM_000051.3)	c.5908C>T	5	P53
ATM(NM_000051.3)	c.1715T>C	5	P53
ATM(NM_000051.3)	c.967A>G	7	P53
ATM(NM_000051.3)	c.5697C>A	9	P53
ATM(NM_000051.3)	c.4577C>T	9	P53
ATM(NM_000051.3)	c.4906C>T	12	P53
CDKN2A(NM_001195132.1)	c.194T>C	1	P53
CDKN2A(NM_001195132.1)	c.262G>T	2	P53
CDKN2A(NM_001195132.1)	c.194T>C	10	P53
CDKN2A(NM_001195132.1)	c.194T>C	12	P53
RB1(NM_000321.2)	c.1544C>T	6	P53
RB1(NM_000321.2)	c.2014G>T	11	P53
TP53(NM_001126114.2)	c.818G>A	10	P53
PIK3CA(NM_006218.2)	c.262C>T	2	PI3K-Akt
PIK3CA(NM_006218.2)	c.2113C>T	2	PI3K-Akt
PIK3CA(NM_006218.2)	c.2119G>A	6	PI3K-Akt
PIK3CA(NM_006218.2)	c.353G>A	7	PI3K-Akt
PIK3CA(NM_006218.2)	c.1624G>A	7	PI3K-Akt
PIK3CA(NM_006218.2)	c.1067T>C	7	PI3K-Akt
PIK3CA(NM_006218.2)	c.2119G>A	7	PI3K-Akt
PIK3CA(NM_006218.2)	c.1035T>A	9	PI3K-Akt
PTEN(NM_001304718.2)	c.435+1G>T	3	PI3K-Akt
PTEN(NM_000314.4)	c.493G>T	6	PI3K-Akt
PTEN(NM_000314.4)	c.640C>T	9	PI3K-Akt
PTEN(NM_001304717.5)	c.927G>A	12	PI3K-Akt
MTOR(NM_004958.3)	c.526G>A	5	PI3K-Akt
MTOR(NM_004958.3)	c.6577C>T	12	PI3K-Akt
APC(NM_001354895.2)	c.1500T>A	1	Wnt

APC(NM_001354895.2)	c.706C>T	3	Wnt
APC(NM_001354895.2)	c.1624C>T	3	Wnt
APC(NM_001354895.2)	c.1658G>A	4	Wnt
APC(NM_001354895.2)	c.4508C>A	4	Wnt
APC(NM_001354895.2)	c.471G>A	6	Wnt
CTNNB1(NM_001904.4)	c.1040T>C	4	Wnt
CTNNB1(NM_001904.4)	c.1628C>T	5	Wnt
CTNNB1(NM_001904.4)	c.1955C>T	12	Wnt
GNAS(NM_080425.3)	c.2531G>A	1	cAMP
GNAS(NM_080425.3)	c.2530C>T	3	cAMP
GNAS(NM_080425.3)	c.2531G>A	5	cAMP
GNAS(NM_080425.3)	c.2531G>A	9	cAMP
GNAS(NM_080425.3)	c.2531G>A	11	cAMP
GNA11(NM_002067.5)	c.196G>A	5	cAMP
GNA11(NM_002067.5)	c.412G>T	11	cAMP
GNA11(NM_002067.5)	c.785C>A	12	cAMP
GNAQ(NM_002072.5)	c.303C>A	2	cAMP
ARID1A(NM_006015.4)	c.5975C>T	5	Chromatin
ARID1A(NM_006015.4)	c.3219G>A	5	Chromatin
ARID1A(NM_006015.4)	c.3562C>T	5	Chromatin
ARID1A(NM_006015.4)	c.4024C>T	5	Chromatin
ARID1A(NM_006015.4)	c.880A>C	6	Chromatin
ARID1A(NM_006015.4)	c.5372C>A	12	Chromatin
IDH1(NM_001282387.1)	c.473C>T	2	EpigeneticRegulation
IDH1(NM_001282387.1)	c.481G>A	6	EpigeneticRegulation
IDH1(NM_001282387.1)	c.394C>T	8	EpigeneticRegulation
IDH1(NM_001282387.1)	c.1004C>T	9	EpigeneticRegulation
KMT2A(NM_001197104.1)	c.5383G>A	4	EpigeneticRegulation
KMT2A(NM_001197104.1)	c.5755G>A	12	EpigeneticRegulation
FBXW7(NM_001349798.2)	c.1160T>A	10	Notch
NOTCH1(NM_017617.5)	c.7171C>T	12	Notch
BRCA2(NM_000059.3)	c.3853G>T	7	DNARepair
SMAD4(NM_005359.5)	c.538C>T	12	TGF-Beta
